# A scoping review on how field epidemiology training programs are addressing regional and global health priorities

**DOI:** 10.3389/fpubh.2024.1490125

**Published:** 2024-12-18

**Authors:** Stephen Leshan Koyie, Marion Muehlen, Navneet Dhand, Anne Perrocheau

**Affiliations:** World Health Organization, Geneva, Switzerland

**Keywords:** epidemiology, public health, global health, health workforce, field epidemiology and laboratory training programs

## Abstract

**Introduction:**

Recent global health events underscore the critical need to strengthen public health capacity worldwide, with epidemiologists playing a key role in disease management at the population level. The international community has recognized the importance of enhancing the public health workforce, including epidemiology capacity. This scoping review explores how Field Epidemiology Training Program (FETP) trainees and graduates have been engaged by their respective health ministries to address public health threats.

**Methods:**

A literature search was conducted in electronic databases (Web of Science, PubMed, and Google) using specific keywords such as “Epidemiologist,” “Field Epidemiologist,” and “Health workforce.” The search focused on English-language articles published between January 2012 and December 2021. Relevant articles were analyzed descriptively, and data on FETP engagement, impact within health ministries, and career paths were extracted.

**Results:**

The search yielded 30 studies from various regions globally. FETP graduates and trainees were actively engaged in national health priorities, including COVID-19 responses such as surveillance, rapid response teams, and case investigations. FETPs have significantly contributed by developing surveillance systems, investigating outbreaks, and responding to natural disasters. Many FETP graduates have assumed leadership roles in Ministries of Health, NGOs, and international organizations.

**Conclusion:**

FETP graduates are integral to priority public health programs and have significantly strengthened public health systems worldwide. Their contributions highlight the importance of investing in the public health workforce, including field epidemiology training, to effectively detect and respond to emerging outbreaks. Further research is needed to assess the long-term impact of FETP graduates on public health.

## Introduction

Recent global health events like the Covid-19 pandemic, recurring Ebola outbreaks, tuberculosis, HIV, and the anticipated impact of climate change on health underscore the need to enhance public health capacity and allied sectors globally. Improved public health capacity helps in early detection of health events, preventing small events from becoming large-scale emergencies and helps to manage essential health services (1). Field epidemiology, a branch of Public Health, is crucial in understanding disease evolution and course and tracking its progress against control measures. The role of field epidemiologists was reinforced during the Covid-19 pandemic response.

The 73rd World Health Assembly recognized the importance of strengthening the public health workforce, especially epidemiologists, in response to the pandemic. The adoption of resolution 73.8.12 emphasized providing appropriate remuneration, resources, and training to health professionals, including under-represented cadres like epidemiologists. The Independent Panel for Pandemic Preparedness and Response also recommended investing in the frontline public health workforce to quickly detect and respond to emerging outbreaks (2). The Intergovernmental Negotiating Body, tasked with developing a pandemic treaty, highlighted the need to invest in the public health workforce, including epidemiology capacity. In December 2022, the INB recommended strengthening and sustaining an adequately skilled and committed health workforce, including at the frontline of pandemic prevention, preparedness, response, and recovery (1).

Despite the crucial role of epidemiologists, there is no International Labour Organization (ILO) classification or job definition for them in many national health systems. Field epidemiology training programs (FETPs) provide on-site training to enhance public health surveillance and response to health threats (3). These programs, structured at three levels—frontline (3 months), intermediate (9–12 months), and advanced (20–24 months) (4)have trained over 22,000 people across 80 countries by 2023 (5). Training is competency-based, combining practical service provision with theoretical courses, with mentors guiding trainees’ professional development.

FETPs have significantly contributed to responding to major public health threats like the COVID-19 pandemic. Although traditionally focused on infectious diseases, FETP graduates and trainees have also worked on non-communicable diseases and responses to natural disasters and humanitarian emergencies. Graduates are expected to assume positions within their national public health systems and leadership roles in ministries of health, NGOs, and international organizations. However, few studies have documented the nature and scope of FETP contributions to public health (6, 7). The main objective of this review is to describe the integration of FETP trainees and graduates into priority public health programs.

### Objectives

1. Review existing literature on the engagement of FETP graduates and trainees in national health priorities.
2. Describe the diversity of activities and functions covered by FETP graduates and trainees.

## Methods

We conducted an electronic search using the Web of Science, PubMed, and Google databases. A combination of Medical Subject Heading (MeSH) terms, subject headings, keywords, and Boolean operators was used to cover concepts related to field epidemiology.

In PubMed, we used search terms like “Epidemiologist,” “Field Epidemiologist,” “Public health,” “Health workforce,” “Field epidemiology,” “Training program,” and “FETP.” These were used both together and separately in various combinations to ensure comprehensive coverage. We applied various combinations such: “Epidemiologist” AND “Field Epidemiologist,” “Public health” AND “Health workforce,” and “Field Epidemiologist” AND “Public health.” “Epidemiologist” AND “Health workforce” (public health OR health workforce) AND FETP training.

For the Web of Science, we used the same terms as in PubMed, with search strings such as: “FETP workforce” AND “Public health,” “Public health” AND “training,” and “Field Epidemiologist” AND “careers.”

Google was used to identify gray literature to fill gaps in the databases. Search terms included “public health, workforce,” combined with one of the following: “training, retention, careers, field epidemiologist.” We sought journal articles, review papers, research reports, and conference reports published in English between January 2012 and December 2021 with full text available, including journal articles, review papers, research reports, and conference proceedings, with available full text. We excluded articles not in English, those unrelated to the epidemiology workforce or FETP, and those without full-text access.

Duplicates were removed, and we screened titles and abstracts for relevance. If abstracts did not provide sufficient information for eligibility, we retrieved the full articles to make an informed decision on inclusion. The review focused on three thematic areas:

1. FETP graduates’ contribution to public health functions.
2. Impact of FETP training programs on public health services.
3. Coordination of surveillance and response activities by the epidemiology workforce.

We developed a standardized data extraction form to capture study characteristics and thematic content relevant to the research questions. Data extracted included publication details (author, year, country/location), thematic focus, type of publication, and the reported engagement of FETP graduates and trainees within ministries of health or other public health organizations.

**Figure 1:**
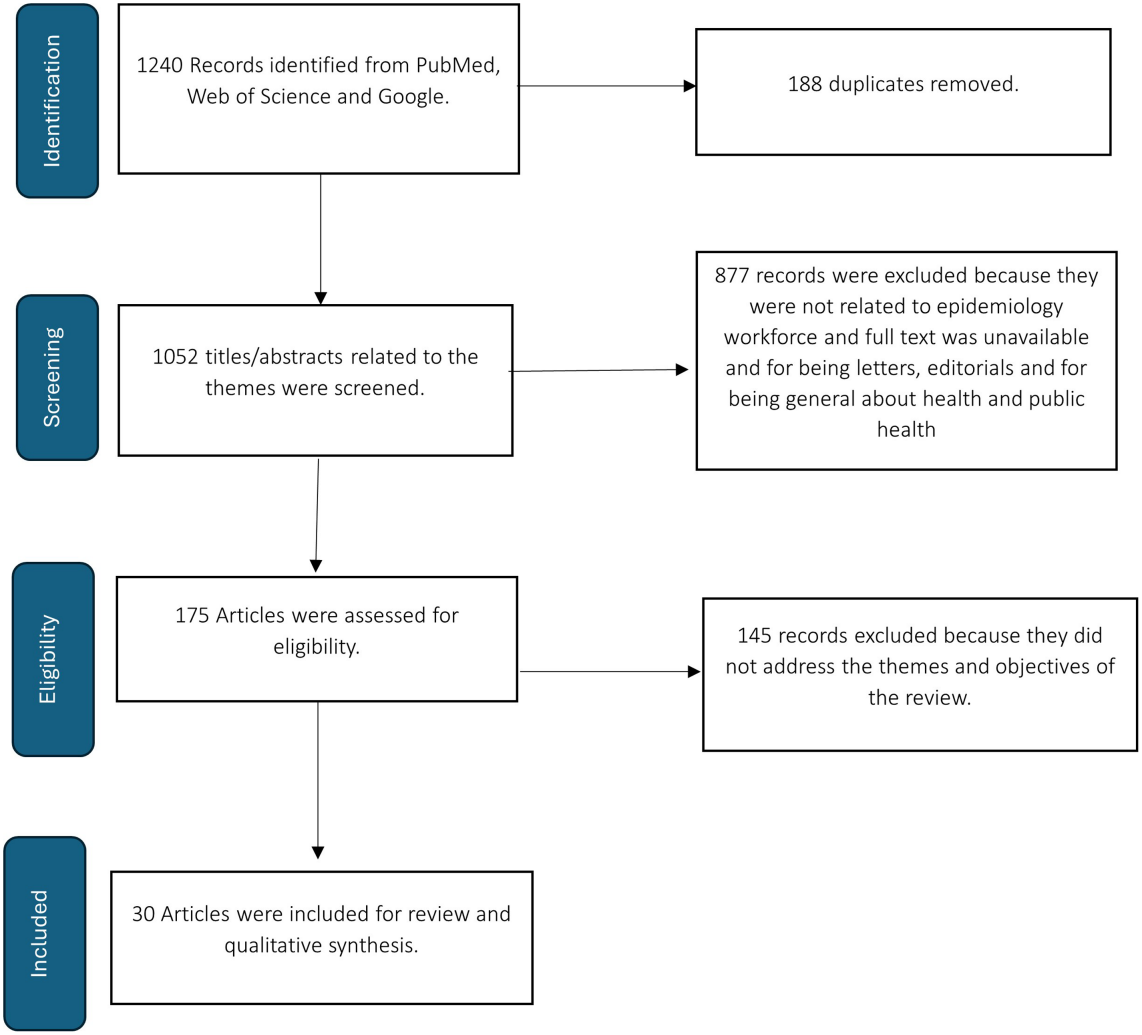
Flow chart showing studies selection for the mini-review.

We conducted a descriptive analysis of publication geographic distribution. We then described the contributions and roles of FETP graduates based on their specific countries engagement within health ministries and other public health bodies. We described the engagement of FETP graduates and trainees in ministries of health and the main areas where they work. The method for selecting articles is presented in Figure 1.

## Results

**Figure 2:**
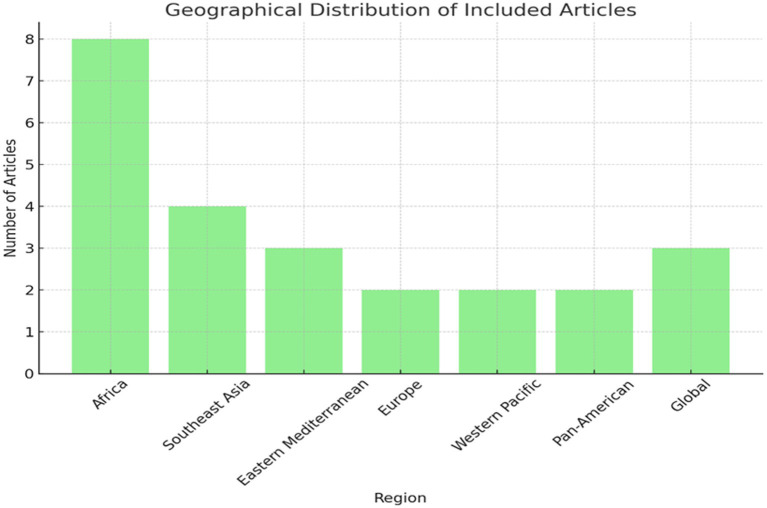
Distribution of articles reviewed by region.

The database search yielded 1,240 results (489 in PubMed, 742 in Web of Science), and a secondary search using Google added nine more results (Figure 1). After screening titles and abstracts based on the inclusion and exclusion criteria, 175 articles were selected for full-text examination. Finally, 30 articles were included in the review (Table 1). Of these, eight were from Africa, four from Southeast Asia, three from the Eastern Mediterranean, two each from Europe, the Western Pacific, and the Pan-American regions, while three focused on a global perspective and were not region-specific (Figure 2).

**Table 1:** Summary table of reviewed articles.

Title of the publication	Authors and reference number	Year	Location
Mozambique field epidemiology and laboratory training program: a pathway for strengthening human resources in applied epidemiology	Baltazar et al. (12)	2017	Mozambique
Yemen field epidemiology training program: a tool for strengthening the public health workforce	Al Serouri et al. (23)	2018	Yemen
Strengthening the global one health workforce: Veterinarians in CDC-supported field epidemiology training programs	Seffren et al. (35)	2022	All FETP Programs supported by CDC
Field epidemiology training programs contribute to COVID-19 preparedness and response globally	Hu et al. (8)	2022	TEPHINET and CDC FETPS
Evaluation of Advanced Field Epidemiology Training Programs in the Eastern Mediterranean Region: A Multi-Country Study	Al Nsour et al. (21)	2021	EMRO
Evaluation of the first two Frontline cohorts of the field epidemiology training program in Guinea, West Africa	Collins et al. (33)	2022	Guinea
Field Epidemiology Training Program Response to COVID-19 During a Conflict: Experience From Yemen	Al Serouri et al. (22)	2021	Yemen
Building Global Epidemiology and Response Capacity with Field Epidemiology Training Programs	Jones et al. (3)	2017	Global
Sustainability of a field epidemiology and laboratory training program: the Ghanaian story	Bandoh et al. (29)	2019	Ghana
The United Kingdom Field Epidemiology Training program: meeting program objectives	Dey et al. (38)	2019	UK
Zambia field epidemiology training program: strengthening health security through workforce development	Kumar et al. (15)	2020	Zambia
Four years into the Indian ocean field epidemiology training program	Halm et al. (27)	2017	Indian Ocean
South Africa field epidemiology training program: developing and building applied epidemiology capacity, 2007–2016	Reddy et al. (28)	2019	South Africa
Strengthening Global Health Security Through Africa’s First Absolute Post-Master’s Fellowship Program in Field Epidemiology in Uganda	Ario et al. (25)	2018	Uganda
Mozambique Field Epidemiology and Laboratory Training Program as responders workforce during Idai and Kenneth cyclones: a commentary	Baltazar and Rossetto (13)	2020	Mozambique
Seven years of the field epidemiology training program (FETP) at Chennai, Tamil Nadu, India: an internal evaluation	Bhatnagar et al. (36)	2012	India
Lessons from the first 6 years of an intervention-based field epidemiology training program in Papua New Guinea, 2013–2018	Ropa et al. (37)	2019	Papua New Guinea
Central America Field Epidemiology Training Program (CA FETP): a pathway to sustainable public health capacity development	Lopez, A; Caceres, VM	2012	Central America
Strengthening national, regional and global health capacity through the WHO Western Pacific Region’s Field Epidemiology Fellowship program	Togami, E; Lowbridge, C; Chinnayah, T; Kato, M; Fukusumi, M; Gwack, J; Matsui, T; Olowokure, B; Li, AL	2021	WPRO
Building public health capacity through India epidemic intelligence service and field epidemiology training programs in India	Singh, SK; Murhekar, M; Gupta, S; Minh, NNT; Sodha, SV	2021	India
Epidemic Intelligence Service Alumni in Public Health Leadership Roles	So, M; Winquist, A; Fisher, S; Eaton, D; Carroll, D; Simone, P; Pevzner, E; Arvelo, W	2022	USA
Time, place, and people: composition of the EPIET Alumni Network and its contribution to the European public health resource in 2013	Pezzoli, L; Keramarou, M; Ladbury, G; Jaramillo-Gutierrez, G; Williams, CJ; Le Menach, A	2014	Europe
An evaluation of the global network of field epidemiology and laboratory training programs: a resource for improving public health capacity and increasing the number of public health professionals worldwide	Subramanian RE, Herrera DG, Kelly PM.	2013	TEPHINET
Epidemic Intelligence Service Officers and Field Epidemiology Training Program in Korea	Kwon GY, Moon S, Kwak W, Gwack J, Chu C, Youn SK.	2013	Korea
Field Epidemiology and Laboratory Training Program, Where Is the L-Track?	Gatei W, Galgalo T, Abade A, Henderson A, Rayfield M, McAlister D, Montgomery JM, Peruski LF, Albetkova AA.	2018	Kenya
Jordan Field Epidemiology Training Program: Critical Role in National and Regional Capacity Building	Al Nsour M, Iblan I, Tarawneh MR.	2018	Jordan
Lessons from the first 6 years of an intervention-based field epidemiology training program in Papua New Guinea, 2013–2018	Ropa B, Flint J, O’Reilly M	2019	Papua New Guinea
Training and Service in Public Health, Nigeria Field Epidemiology and Laboratory Training, 2008–2014			Nigeria

In this review, we use the term “trainee” to designate a person enrolled in an FETP and “graduate” for a trainee who has completed the program.

### Engagement of FETP trainees in national health priorities

In 2022, the Training Programs in Epidemiology and Public Health Interventions Network (TEPHINET), a global network of field epidemiology programs, conducted a study in which 74% 65/88 of FETPs across all WHO regions reported that trainees and graduates were engaged in COVID-19 response activities, such as surveillance, rapid response teams, case investigations, country-level coordination, and risk communication. All these activities require competencies that FETPs are designed to address (8, 9) (Table 2).

According to TEPHINET’s annual surveys, since 1980, over 18,000 FETP graduates have been trained across the program’s three tiers. During their training, participants evaluated, developed, or implemented more than 8,680 disease surveillance systems, investigated over 14,190 outbreaks or acute health events, delivered over 11,250 oral and poster presentations at scientific conferences, and published more than 3,710 peer-reviewed articles from TEPHINET member FETPs (10).

**Table 2:** Summary table of the activities by FETP trainees and graduates in different countries.

Major FETP activities per Country	
Country	Major activities
Mozambique	Outbreak investigations, polio campaigns, disaster response
Uganda	Outbreak response unit, reports to National Task Force
Nigeria	Investigation of environmental lead poisoning, cholera
Namibia	One-health approach training, zoonotic disease response
Jordan	Sentinel surveillance for foodborne illness, MERS-CoV
Yemen	Outbreak investigations, COVID-19 response, MERS-CoV
Philippines	Development of surveillance systems, disaster response
Zambia	Typhoid, anthrax investigations, HIV and malaria improvements

FETPs graduates career paths per region	
Region	Common roles
Americas	CDC directors, state epidemiologists, regional consultants
Africa	Epidemiologists, program managers, field supervisors
Asia	Public health supervisors, outbreak investigators
Europe	Directors of national PHIs, roles in WHO and ECDC

#### In Asia

Flint et al. (6) highlighted the impact of FETPs in ministries of health. In the Philippines, FETP graduates and trainees developed surveillance systems for acute infectious diseases, later expanding to cover human immunodeficiency virus (HIV) seroprevalence, behavioral risks, acute flaccid paralysis, and fireworks-related injuries. During major disasters, the Philippine Department of Health used the FETP network for rapid health assessments and risk evaluations, actively monitoring over 100,000 evacuees and reporting to disaster managers and the Cabinet (6, 11).

#### In Africa

In Mozambique, although the FELTP is relatively new, it has significantly strengthened the health system. Graduates and trainees conducted outbreak investigations, monitored mass polio vaccination campaigns, evaluated malaria bed-net distribution, conducted event based surveillance during mass gatherings and responded to natural disasters like floods (12). During the Idai and Kenneth cyclones, they carried out rapid health assessments of displaced populations, implemented early warning systems, and developed tools for investigating epidemic-prone diseases (13).

In Uganda, the Public Health Fellowship Program (PHFP) formed and now leads the Department of Health’s outbreak investigation and response unit. Trainees regularly submit outbreak reports to the Public Health Emergency Operation Center and present them at the National Task Force for Epidemic Preparedness and Response, aiding in outbreak prevention and control nationwide (14).

The Zambia Field Epidemiology Training Program (ZFETP) has played a crucial role in outbreak investigations, including typhoid fever, anthrax, and less known incidents like konzo, a neurological disease from cassava poisoning. Their evaluations of HIV and malaria surveillance systems led to significant improvements (15).

In Nigeria, graduates have been involved in investigating complex outbreaks of environmental lead poisoning, cholera, meningitis, Lassa fever, and chemical poisoning from a contaminated teething mixture, as well as investigating measles, rabies, and leptospirosis outbreaks (16–18). Within 6 years, they investigated 133 suspected outbreaks, ranging from environmental-related diseases to neglected tropical diseases (18).

Namibia’s advanced FELTP trains professionals from various fields in the one-health approach, enabling effective responses to zoonotic disease outbreaks, such as the recurrent CCHF outbreaks and anthrax among hippos in Bwabwata National Park (19).

#### In Middle East

In Jordan, FETP trainees and graduates established a sentinel surveillance system for foodborne illness and played a key role in investigating the MERS-CoV outbreak (20). Evaluation of the FETP found that graduates in the Eastern Mediterranean region have been a vital resource in tackling public health challenges and in strengthening their countries’ public health systems through surveillance, data analysis, training, and outbreak response (21).

The FETP has been instrumental in supporting Yemen’s health system in the COVID-19 response throughout the war and the current crisis and emergency situations, as the security situation deteriorated. Because access to some areas was limited for international support, the trainees and graduates have been extremely valuable in supporting the activities of the Ministry of Public Health and Population (22). Yemen’s FETP trainees and graduates played an active role in investigating and responding to over 100 outbreaks, including cholera, dengue, rabies, Neisseria meningitis, measles, pertussis, hepatitis, and food poisoning. The trainees demonstrated capacity for prompt responses to outbreak detection, rigorous investigation reports, and provided recommendations for containing and preventing future outbreaks. In addition, FETP trainees identified and documented the first-ever outbreaks of Chikungunya virus, West Nile virus, and Middle East respiratory syndrome coronavirus (MERS-CoV) reported in Yemen (23).

### FETP graduates’ roles and career paths

Although the reviewed literature did not specifically explore the career paths of FETP graduates, efforts were made to identify roles and positions held by alumni/graduates. Key responsibilities highlighted include designing and implementing public health surveillance systems, investigating and controlling disease outbreaks, and evaluating different public health activities and programs (3, 8, 24, 25). Some studies have estimated retention rates of FETP graduates within national health systems (6, 23), but details on those working in public health NGOs, international organizations, and in which capacities they serving are often not provided.

Alumni of FETPs have advanced to positions of leadership in ministries of health, non-governmental and international organizations, and other health organizations (3, 8, 24). However, the actual proportion of graduates serving in specific capacities remains unclear.

#### In the Americas

In the Unites States of America, a study by Marvin et al. found that four out of 12 CDC directors from 1953 to 2016 were EIS graduates, who led the agency for almost 25 years. EIS graduates also accounted for 58% of the 50 CDC center directors between 2000 and 2016, with state epidemiologists being the largest leadership group accounting for 61 (35%) of the 175 participants (24). In some countries, FETP alumni hold leadership positions such as Permanent Secretaries in Ministries of Health, Program Directors for epidemiology, surveillance, and specific disease control programs while others take positions with WHO (e.g., national professional officers) and other non-governmental public health–associated organizations (3).

In Central America, 80% of FETP graduates continue to work in their country’s Ministry of Health, 5% in international roles, and 15% in other organizations (26). Some alumni lead national epidemiology offices or work with CDC as regional consultants in the Global AIDS Program and Avian Influenza Program (17).

#### In Africa

All graduates in Mozambique were assigned to the national health system through various programs at the central and provincial levels, the National Institute of Health reference labs, and the Ministry of Defense (Military Hospital Laboratory of Maputo) (12).

The Indian Ocean FETP graduates work in national or regional government positions, mostly promoted after finishing the program, Health Surveillance Unit of the Indian Ocean Commission, Pasteur Institute in Madagascar, and World Health Organization office in Madagascar (27).

In South Africa, some FETP graduates work for Africa CDC, while 58% are employed by South African government organizations, either at the national (32%) or provincial (26%) level. Additionally, (27%) are employed by non-governmental groups (NGOs) (28).

In Uganda, some of the graduates have been retained by their FETP as field supervisors, some are employed by WHO in countries like South Sudan, while others are serving as field epidemiologists with the African Field epidemiology Network (AFENET). They have all joined the National Rapid Response Team, which is responsible for preparedness and response to outbreaks. The Ministry of Health is actively seeking to employ more PHFP graduates to fill permanent field epidemiology positions in a range of high-priority programs (25).

A study conducted in Ghana in 2019 indicated that about 20% (14/70) of graduates were not placed anywhere in the Health Service after completion of the program. However, in the same study, 30% (20/70) of the alumni occupied positions as Directors of Public Health Service in the Ministry of Health, Ghana Health Service, and Veterinary Services Department. Twenty percent (14/70) also lead various departments in the health facilities (29).

In Kenya and Tanzania, graduates, were reported to be working as epidemiologists, program managers, program coordinators, or regulatory/inspection boards. Professional upward mobility was high; 87% (Kenya) and 73% (Tanzania) graduates reported promotions after FETP graduation either in the same or in new institutions (30).

#### In Europe

The EPIET alumni network (EAN) in Europe performed a study reporting that 94/155 (61%) of graduates identified public health epidemiology as their job status, followed by academic. One-third 53/155 (34%) of respondents said they worked at national public health institutes (PHIs), followed by international PHIs (including the ECDC), regional PHIs, and research institutions (31). EPIET graduates have gone on to key positions of leadership in international organizations like WHO, become directors of regional CDCs, like the European and the Gulf CDC, directors of NPHIs, and held key positions in MoHs, among others.

## Discussion

This study summarizes the involvement of FETP graduates and trainees in regional and national public health priorities and their career trajectories. The literature review highlights the significant global impact of FETPs in strengthening public health responses, particularly during the COVID-19 pandemic and other disasters. Across all six WHO regions, FETP trainees and graduates have been actively engaged in various aspects of the pandemic response, including surveillance, rapid response teams, case investigation, coordination, risk communication, and community engagement. These activities align with the core competencies that FETPs aim to achieve, demonstrating the effectiveness of these programs in preparing professionals for real-world public health challenges (13–15, 20, 22).

Our findings indicate that FETP alumni have engaged in various domains outlined in the Essential Public Health Functions for International Health Regulations implementation (6, 8, 18, 21, 32, 33). This positions FETPs at the forefront of developing a public health workforce capable of implementing IHR and other public health functions.

Ministries of Health partner with FETP programs and acknowledge the contributions of FETP graduates and trainees to public health systems. This is evidenced by the extensive engagement of FETPs at all levels, from national to district, the adoption of surveillance systems developed by graduates, the acceptance of FETP recommendations, and the promotion of graduates to leadership positions within ministries of health (3, 8, 19, 24). Nonetheless, some countries still feel the program lacks sufficient recognition.

The career trajectories of FETP graduates highlight the long-term impact of these programs on public health. Whether in government ministries, international organizations, NGOs, or academia, FETP alumni have assumed leadership roles and significantly contributed to public health policy, research, and practice. The retention of graduates within national health systems, as seen in Mozambique and South Africa, underscores the value placed on FETP-trained professionals and their ongoing contributions to public health. However, challenges remain in tracking alumni career paths to assess their long-term impact on health outcomes, which is crucial for informing program improvements and resource allocation.

This review contributes to the FETP Enterprise, a vast multi-partner effort in developing, implementing, and evaluating FETPs in over 100 countries. It highlights the contributions of FETP graduates and fellows across all public health domains, advocating for enhanced field epidemiology capacity globally. Additionally, the University of Newcastle, Australia, is conducting operational research on the health impact and economic value of PH activities involving FETP graduates (surveillance, outbreak investigations, interruption of transmission chains) (34). This work is essential for advocating for more efficient government investments in FETPs.

WHO is leading efforts to develop a Roadmap for the Public Health and Emergency Workforce, aiming to map workforce capability in 20–30 countries over the coming years. This initiative seeks to better define and classify key public health roles, ensuring a professional public health workforce with a clear career structure to attract and retain well-trained staff.

WHO has recently established a unit for Field Epidemiology Capacity Strengthening with the objective of initiating efforts in Regional and Country Offices towards supporting FETPs or field epidemiology capacity strengthening programs. The new unit will lead in developing a global roadmap that ensures the pressing need to establish field epidemiology as a fundamental component of the public health workforce. Together with global and regional associations of FETPs, the skills and competencies of FETP need to be defined and standardized and incorporated within a new occupation classification with ILO, as part of the roadmap. Further efforts in this area should collect example job descriptions in order to create a repository of the roles that FETP graduates can play to ensure essential public health functions. National Governments, National Public Health Institutes, and Public Health Schools and Training Institutions need to collaborate to develop epidemiology as a career with a clear professional structure. Some literature suggests the need for the establishment and implementation of strategies that will ensure the retention of FETP graduates by appropriately placing them where their skills are needed, and providing them with remuneration incentives (3).

While current FETPs in most countries have been constructed upon the model of the Epidemic Intelligence Service of the US CDC, more curricula are being developed to reinforce field epidemiology workforce health emergency responses. This will complement the existing national FETPs and extend the number of field epidemiologists in countries and for international deployments. As we need to equip the future field epidemiologist with the skills and competencies to face the health challenges that lie ahead, applying the One Health approach to face challenges like zoonosis, vector-borne diseases, climate change, and others is an important next step. Competencies in One Health field epidemiology should be added to current FETP curricula as they are relevant to the response to complex emergencies, humanitarian health, migrants’ health, famine, drought, and other climate change related threats.

Some limitations are inherent to the methodology applied: results of literature search are dependent on the acceptance of the manuscripts by scientific journals. Negative results, such as a poor retention rate of FETPs or the poor performance of FETP graduates in an outbreak investigation, are less likely to be published. Also, we noted that authors of most of the studies reported in this review are affiliated to institutions that are supporting FETPs across the world, either through funding, developing training materials or mentoring the FETP trainees. At the same time, we can see that all papers systematically describe success stories, which tend to prove that in some instances there might be a need to convince others of the success of the work done. This might be seen as a bias derived from a conflict of interest not always reported in the articles.

A thorough examination of the challenges FETP graduates encounter when implementing public health interventions was hindered by limited data availability. Certain regions may experience distinct obstacles to integrating FETP graduates into their national health systems. However, the data collected lacked the regional specificity needed to fully explore these unique barriers. Thus, further studies are recommended to investigate this issue in greater depth.

While FETP graduates’ contributions to capacity building and response activities were well-documented in the reviewed literature in this study, direct measurements of health outcomes—such as reduced outbreak frequency or improved response efficiency—were rarely reported. This gap in outcome-based evaluations underscores the need for future research to quantify the public health impact of FETP graduates and provide a stronger evidence base for assessing program effectiveness.

## Conclusion

FETPs have emerged as indispensable tools for strengthening public health systems, enhancing epidemic preparedness and response, and building a cadre of skilled professionals capable of addressing evolving health challenges. The evidence presented in this literature review highlights the importance of continued investment in FETPs and the need for ongoing research and evaluation of the FETP programs to maximize their impact on global health security. The review shows that the engagement of FETP trainees and graduates encompasses the traditional epidemiology domain of surveillance and outbreak investigation and is now extending to other public health issues such as non-communicable diseases, climate change, and road accidents. FETPs around the world shall be better supported and included into the Public Health systems of their countries if they are recognized and their occupation is defined in the ILO classification.
